# Shale Gas Extraction in North Carolina: Research Recommendations and Public Health Implications

**DOI:** 10.1289/ehp.1307402

**Published:** 2013-10-01

**Authors:** Adrian Down, Martin Armes, Robert B. Jackson

**Affiliations:** 1 Nicholas School of the Environment and Center on Global Change, Duke University, Durham, North Carolina, USA; 2 Research Triangle Environmental Health Collaborative, Raleigh, North Carolina, USA

North Carolina has no history of large-scale commercial oil and gas extraction, and the state’s legislative framework for regulating drilling was, until recently, based on laws passed in the 1940s. However, areas of the state are likely to undergo horizontal drilling and hydraulic fracturing for natural gas and oil in the near future. North Carolina thus has a unique opportunity to produce a legislative framework that *a*) incorporates experiences from other states, *b*) includes state-of-the-art technologies and best practices, and *c*) protects the health of North Carolina’s citizens and ecosystems.

Knowledge of the health risks associated with hydraulic fracturing is sparse. Some of the chemicals that can be used in the hydraulic fracturing process are toxic (Bamberger and Oswald 2012; Colborn et al. 2011). However, the concentrations of these chemicals used at a given well site are not disclosed in most states; thus, evaluating the risk of exposure and associated health impacts is difficult. Residents living < 1 km from hydraulically fractured wells are potentially at greater risk for health effects from natural gas development, which in some cases may include exposure to trimethylbenzenes, xylenes, and aliphatic hydrocarbons in air (McKenzie et al. 2012). These residents can also, but do not always, have higher concentrations of dissolved methane and other gases in their drinking water (Jackson et al. 2013; Osborn et al. 2011; Warner et al. 2013).

In October 2012, the Research Triangle Environmental Health Collaborative convened a summit of experts from the oil and gas industry, nonprofit organizations, government agencies, and academia to consider the potential impacts of horizontal drilling and hydraulic fracturing in North Carolina. The summit included three working groups that focused on potential outcomes related to hydraulic fracturing: exposure pathways, health impacts, and social impacts. The summit recommended actions and policies to safeguard the health of North Carolina’s citizens and environment if hydraulic fracturing occurs in the state (Research Triangle Environmental Health Collaborative 2013). The recommendations should also be useful for policy makers in other states.

Summit participants discussed numerous recommendations, with three categories having the broadest support. First, the participants noted the importance of collecting comprehensive background data on air quality, water quality, and human and ecosystem health before oil or gas drilling occurs. Such data provide a baseline documenting current conditions that can be used to determine whether changes take place in the future, thus protecting both citizens and drilling companies from unfounded claims of damages. The quality and quantity of ground and surface water resources potentially affected by drilling should be studied, including analyses of major ions, trace metals, dissolved gases such as methane, radioactivity, and a range of organic compounds. Hydrocarbons from oil and gas wells should be characterized based on chemical and isotopic composition, which aids wastewater treatment and makes it easier to identify potential contamination if hydrocarbons are released into the environment. Ambient air monitoring of potential drilling areas should be performed because emissions from drilling sites may contain volatile organic compounds, particulates, and other contaminants. Ecosystem health, such as the identity and abundance of stream organisms, in the areas near drilling should also be assessed.

Second, participants supported a comprehensive health impact assessment (HIA) as a means to monitor and avoid potential health problems in the future. An HIA should combine local-, regional-, and state-level medical and demographic data. Tracking of any health problems encountered in other states with hydraulic fracturing could provide early warning of heath problems that might occur in North Carolina and allow preventative action. Psychological and other stressors beyond direct chemical exposure should be considered, including sources such as increased road traffic and light and noise pollution. The HIA should also examine the potential economic costs associated with health impacts, including, for example, potential water remediation or increased rates of asthma.

The third broad recommendation was to create a community needs and assets assessment (CNAA) to address potential social impacts. The CNAA should *a*) identify what jobs will be available to local workers, *b*) develop citizen stakeholder forums and reporting mechanisms, *c*) update transportation planning and safety training, and *d*) implement strong consumer protections. The working group on social impacts also recommended creating an ombudsman to facilitate communication between stakeholders and industry.

For all three of these recommendations as well as for the many others included in the report (Research Triangle Environmental Health Collaborative 2013), it is important to clarify who is responsible for collecting such data and how to pay for it. One mechanism to ensure access for background data collection is to make gas well permits contingent on temporary site access for ambient air and water monitoring before, during, and after drilling and hydraulic fracturing. Policy makers might consider a bonding and remediation program to provide adequate cleanup, remediation, and maintenance funds. The cost of performing comprehensive environmental or health remediation should be considered in assessing bonding fees. Finally, it is important to decide—before drilling begins—how increased costs of infrastructure maintenance and staff will be apportioned.

North Carolina has the opportunity to develop model programs and best-management practices for shale gas drilling and hydraulic fracturing. Our recommendations complement the work of North Carolina’s Mining and Energy Commission and can help North Carolina and other states protect the public’s health in areas undergoing unconventional oil and gas production.
